# ARTISTIC ENGAGEMENT AT A MEMORY CARE FACILITY, VIA A TELEPRESENCE ROBOT

**DOI:** 10.1093/geroni/igac059.2822

**Published:** 2022-12-20

**Authors:** Amy Knepple Carney, Vanessa Hillman, Chloe Lehman, Elizabeth Holland, Lizzie Gowin

**Affiliations:** University of Wisconsin- Oshkosh, Oshkosh, Wisconsin, United States; University of Wisconsin- Oshkosh, Oshkosh, Wisconsin, United States; University of Wisconsin- Oshkosh, Oshkosh, Wisconsin, United States; University of Wisconsin- Oshkosh, Oshkosh, Wisconsin, United States; University of Wisconsin- Oshkosh, Oshkosh, Wisconsin, United States

## Abstract

There is a growing body of research that shows participation in arts interventions (e.g., poetry, storytelling, music, and dance) can be beneficial for those living with dementia. The current study examines social interactions when the artist is not physically present, but in the room via a telepresence robot. Through the telepresence robot, dementia care residents were connected to a network of arts presenters. Using a checklist examining social behaviors (verbal and non-verbal), we assessed residents’ social engagement with the artist, other residents, and staff during these creative visits. Typical activities within the care facility were also observed. The observations were conducted on 10 individuals living within the memory care facility. Although the typical facility activities elicited social verbal behaviors, they tended to be more physical in nature and had a larger number of social nonverbal behaviors. When the behavior was verbal it tended to be more noises of excitement, such as “wooo” and “oh yeah.” Although the artist engagement activities elicited social nonverbal behaviors, they tended to be more verbally engaging and there were a larger number of social verbal behaviors during these activities. With the artist engagement we observed residents spontaneously singing songs, and helping to create poetry. The facility was already using activities that allowed the residents to become physically engaged and with the addition of artist visits there was an increase in verbal engagement. By having greater options for residents to create engagement we may see people who don’t typically engage become more involved.

